# Identifying strategies to improve adverse drug reporting through key informant interviews among community pharmacists in a developing country

**DOI:** 10.1038/s41598-024-67263-8

**Published:** 2024-07-22

**Authors:** Sunday Odunke Nduka, Chiamaka Omelebere Ibe, Mercy Adamma Nwaodu, Chana Chapchet Robert

**Affiliations:** 1 Department of Clinical Pharmacy and Pharmacy Management, Faculty of Pharmaceutical Sciences, Nnamdi Azikiwe University, Awka, Anambra State Nigeria; 2 Procurement and Supply Chain Unit, United Nations Development Programme (Global Fund Project), Harare, Zimbabwe; 3 Department of Clinical Pharmacy and Pharmacy Practice, David Umahi Federal University of Health Sciences, Uburu, Ebonyi State Nigeria

**Keywords:** Adverse drug reaction reporting, Community pharmacists, Key informant interview, Pharmacovigilance, Medicine safety, Health care, Medical research

## Abstract

Reporting adverse drug reactions (ADRs) is fundamental in improving medication safety. Community pharmacists (CPs) being the first point of contact for individuals seeking healthcare in a community, play a significant role in ADR reporting. However, this has been poorly implemented in many countries including Nigeria. This paper aims to explore stakeholders’ perspectives on current reporting practices and suggest ways to enhance ADR reporting among CPs in Nigeria. This qualitative study employed a purposive sampling approach to identify key informants. Key informant interviews (KIIs) were conducted with 25 carefully selected pharmacists, using a semi-structured interview guide between July 2023 and August 2023. The interview transcripts were analyzed using a thematic content approach. While a low ADR reporting trend was observed among all participating pharmacists, it was notably higher among those with less than five years of experience. The main barriers to ADR reporting, as identified by the interviewed community pharmacists were lack of awareness and knowledge, absence of motivation, and insufficient feedback from National Agency for Food Drug Administration and Control (NAFDAC). Training and awareness campaigns were the most frequently suggested methods for improving ADR reporting. Other proposed strategies included providing motivation, regular feedback, establishing mandatory reporting, and simplifying the reporting process. The study has highlighted the suboptimal ADR reporting practices among CPs in Anambra state. It underscores the significance of training, sensitization, advocacy, and other related interventions as pivotal means to enhance ADR reporting in this group. Furthermore, there is a pressing need for intervention-based studies to delve into and implement these approaches effectively.

## Introduction

Adverse drug reactions (ADRs) pose a significant public health challenge, causing substantial morbidity and mortality^1,2^. They result from unintended and harmful responses to medications at typical doses^3^. ADRs not only raise public health concerns but also lead to high hospitalization rates^4,5^, reduced therapeutic effectiveness, and substantial healthcare costs. Developing countries like Nigeria experience higher ADR, ranging from 10 to 30%, compared to developed countries such as the UK, Norway, and France where it’s been reported to be 16%, 11.5%, and 13% respectively^3^. ADRs have adverse impacts on individuals and society at large, with approximately 3–5% of reported hospitalizations globally being attributed to them^6–8^, resulting in some life-threatening situations^9^. In sub-Saharan Africa, serious ADRs rank as the fourth to the sixth leading cause of death in hospitalized patients^10,11^, prolonging hospital stays^5^.

In addition to potential harm to health, ADRs also impose a significant economic burden on nations, estimated at about 30 billion dollars annually in the United States^11^ in 2011 with approximately 197,000 annual deaths in 2008 in Europe^12^.

According to the World Health Organization (WHO), a substantial portion (10–80%) of Adverse Drug Events (ADEs) can be prevented^13^. In Nigeria, preventable ADRs are the primary cause of hospitalization and in-patient morbidity and mortality^14^. Thus, pharmacovigilance, which encompasses the detection, assessment, understanding, and prevention of ADRs, is crucial in mitigating the harm caused by preventable ADRs.

To combat this global issue, the WHO established an International Pharmacovigilance system for ADR monitoring and reporting among member states. In Nigeria, the National Pharmacovigilance Centre (NPC) was established in 2004 under the National Agency for Food Drug Administration and Control (NAFDAC). The NPC relies on spontaneous reports from healthcare professionals and the public, submitted through ADR forms also known as yellow forms or Individual Case Study Report (ICSR) forms, which can be completed manually or reported online.

Healthcare professionals particularly pharmacists play a vital role in contributing to pharmacovigilance data due to their expertise in pharmacotherapy^15^. Community pharmacists in particular as accessible healthcare providers with direct patient interaction^16^, are well-positioned to monitor medication safety and promote pharmacovigilance activities. However, ADR reporting in Nigeria is significantly underreported, with only 1375 ICSRs submitted in 2016, falling far below the expected 34,000 reports needed for adequate reporting^17,18^.

This poor reporting issue persists across various regions of the country and among different healthcare practitioners including community pharmacists^11,15,19–21^. Although other countries in the WHO drug-monitoring program report similar issues, it seems to be more prevalent in Nigeria, presumably due to a heavy reliance on healthcare providers and alternative sources of medications^22^.

Despite efforts to address this, underreporting of ADR remains a persistent problem, necessitating an urgent need for enhanced drug safety surveillance. Meanwhile, stakeholder engagement has been proven to be a valuable tool in generating relevant evidence for real-world healthcare decisions, including the implementation of evidence-based interventions^23,24^. This study therefore aims to assess stakeholders’ perspectives on current reporting trends and ways to improve adverse drug reaction reporting among community pharmacists in Nigeria through interviews.

## Methods

### Study design

This qualitative study employed key informant interviews (KII) to explore stakeholders’ perspectives on ADR reporting among community pharmacists in Anambra state, Nigeria between July and August, 2023.

### Study area and population

Anambra State is located in the Southeastern region of Nigeria and has an estimated population of 6,358,311 as of 2021^25^. With its capital in Awka, Anambra hosts a substantial number of community pharmacists^26^ with more than 150 registered community pharmacists in the state according to information from the Pharmacy Council of Nigeria (PCN), Anambra State office. However, in 2018, the rate of ADR reporting among community pharmacists was observed to be low in some southeastern states^15^.

### Sampling and sample size

A purposive sampling approach was employed in selecting 25 key informants (KIs) including five practicing community pharmacists with over 10 years of experience (PPT), four practicing community pharmacists with less than 10 years of experience (PPF), one past and one present Director of Pharmaceutical Services (DPS) in the state, two participants from the State Ministry of Health (PSMoH), and, one representative of the Pharmacy Council of Nigeria (PCN). Four present (PRE) and four past (PAE) executives of the Association of Community Pharmacists in the state, with three pharmacists in academia (PA), were also included. Participants were selected by creating a list of potential key informants and then, narrowing down the list based on specific criteria: regulatory roles (PCN and PSMoH, including DPSs), practicing community pharmacists and those with leadership experience in the association of community pharmacists in the state. Academic pharmacists were included for their training roles, while young pharmacists (under 10 years of experience) were included to balance the perspectives of the older professionals.

### Instrument development and validation

The study utilized a semi-structured questionnaire which was developed after reviewing previously published works^27,28^ followed by content validation by three experts, and then, pre-tested, and further refined accordingly^29^. The instrument was pilot-tested on 5 community pharmacists who were not part of the study population. At the end of the pilot study, a few amendments were made to clarify some of the questions and to further improve on the instrument. The questions covered areas such as ADR reporting knowledge, reporting channels, barriers to reporting, and strategies for improvement (File S1).

### Data collection and analysis

Data collection involved face-to-face interviews conducted in English Language, lasting 30–45 min each, at the convenience of the key informants. Interviews were recorded with the informants' consent and transcribed according to established protocols^30^. The data were then analyzed using NVIVO software QRS 2011 (https://lumivero.com/product/nvivo/), employing a thematic content approach. Relevant themes were identified through careful analysis of the transcripts, and a data-coding scheme was established. The researchers initially conducted a thematic content analysis to identify key themes. This was followed by coding performed by the researchers and an assistant, with final validation involving the researchers, the assistant, and the supervisor.

The perceived level of involvement of community pharmacists comprised 1 item with a 5-Likert scale of ‘no involvement, very rarely involved, rarely involved, occasionally involved and maximally involved’ scored as ‘0’, ‘1’, ‘2’, ‘3’ and ‘4’ respectively.

### Ethical approval and consent to participate

Ethical approval with the reference number COOUTH/CMAC/ETH.C/VOL1/FN:04/280 dated 01/06/2023 was obtained from the ethics committee of Chukwuemeka Odumegwu Ojukwu Teaching Hospital, Nigeria. Oral informed consent was also obtained from all key informants prior to the interview. All methods were carried out in accordance with relevant guidelines and regulations.

## Results

### Perceived knowledge and involvement in ADR reporting

The 25 Key Informants (KIs) exhibited some commendable level of knowledge and understanding of ADRs. They unanimously emphasized the importance of all healthcare practitioners, including pharmacists in monitoring and reporting ADRs. While acknowledging the critical role of community pharmacists’ involvement in pharmacovigilance activities, reporting within the state was notably deficient with an average rating of 1.56 on a 5-point scale. Eight informants revealed that they had never reported an ADR while seventeen had submitted varying numbers of reports, averaging two reports per participant. They often observed ADRs but preferred to address them directly instead of reporting them. They feel that reporting ADR cases might not always be necessary since they could take action to address it. Some representative comments included:

“Most time I just advised them to withdraw the particular drug I feel may be the cause of the ADR and once withdrawn and the ADR stops, I stop there without reporting”. (PRE3).

“No, I don’t report because sometimes I know what to do to arrest the situation …” (PPT3).

“….. I think that I reported some time ago, some years back, but I think that it is not fully structured and not fully understood by the community pharmacist. So when you observe them you take actions to ameliorate the side effects without necessarily going to the reporting network, through NAFDAC and filling the yellow form……” (PPT1).

However, one KI suggested that some CPs refrain from reporting because they are afraid of disclosing that a patient experienced an ADR due to a medication they provided. They prefer to keep it confidential.

“…I have not reported because I’ve always been skeptical about whom to report the reaction to because when the patient comes and tells you his or her reaction to the drug, what you do is to try and find the remedy to bring down the adverse drug reaction. Reporting it, who do you report to? And even when you know who to report to you wouldn’t want people to know that you gave a patient a drug and the patient reacted to it. It is not an open knowledge thing….” (PAE3).

“…Nobody wants to let people know that he had an adverse reaction, maybe he treated a patient and the person had a hypersensitivity reaction, or the patient slumped. He would not want anybody to know about it. It is like a closely guarded secret. Not that you don’t know how to report and whom to report it to. You don’t want that thing to leave your office….” (PAE3).

One of the KIs, who was once a national executive of ACPN, also mentioned that the distance between his pharmacy and the NAFDAC office poses a challenge to his reporting as he is expected to collect the form from NAFDAC and return it after completion.

“…Before now when the yellow papers were a little bit on the advocacy level, I had reported but sincerely as of now, I have mellowed down on the frequency of reporting. One of the factors is that to get a copy of the yellow form, you need to move distances to go to NAFDAC, which the practice may not allow such time. So if there are spots close to your vicinity where you can collect these forms, it will be very easy for people to do. ….” (PAE1).

Furthermore, negligence was cited by some respondents as one of the attitudes displayed by community pharmacists toward ADR reporting. Two respondents articulated these sentiments:

“…No (reporting), the reason is negligence, it is not like I don’t know that it is the right thing to do but I don’t just feel like doing it….” (PSMoH2).

“…. I have never reported any. I have not really been concerned or even taken note…” (PRE4).

### Previous interventions to improve ADR reporting in the state

There were mixed sentiments regarding interventions previously conducted in the state to enhance ADR reporting. A significant number of the respondents indicated that they were not aware of any specific training or interventions aimed at improving ADR reporting in the state. One respondent stated:

“…. I have not actually been opportune to be in any training if there is any. In the past, I have not heard about any training for such. I believe that if such training is organized it would go through the Association of Community Pharmacists in the State. So that all community pharmacists would be aware that it is ongoing….” (PRE1).

However, some respondents mentioned that they had conducted interventions in their respective areas of practice. For instance, one of the respondents who presently works in a hospital affirmed that those who worked under him received training on ADR reporting. She ensured they passed through the pharmacovigilance unit to acquire the necessary knowledge.

“…. All the people that trained under me passed through the unit. There we have a unit for pharmacovigilance, drug information unit ….” (DPS1).

Another respondent, who had worked in the State Ministry of Health and presently works in a hospital, mentioned that she had personally trained some other pharmacists and interns on ADR reporting.

“…. I personally do clinical meetings on Tuesdays and I train interns on Thursdays. At one of those clinical meetings, I printed a yellow card. I taught them and went to show it to them. After that training, I enjoined all to partake and do the needful. It was after that training that one intern got me an ADR report. …..” (PSMoH2).

Other respondents also shared:

“……I don’t know about community pharmacists but as a lecturer. I make sure that I expose the students very well to the different types of ADR, and the reporting procedure and also tell them not to expect any type of monetary reward or whatever but to have that sense of commitment to duty. It is a call to duty……” (PA1).

“….. Yes, we went through training as community pharmacists then by NAFDAC and that should be about 15 years ago. We went through training on adverse drug reporting, that was then but recently there hasn’t been any formal training instituted. I even think that through Mandatory Continuing Professional Development (MCPD), that session of MCPD should be handled by NAFDAC where they will introduce it and have these discussions….” (PPT4).

A summary of the key themes and the subthemes as identified in the KI interviews is presented in Table 1.

**Table 1:** Summary of key themes and subthemes from the KII.

Themes	Subthemes
Barriers to ADR reporting among community pharmacists	Lack of knowledge and awareness Lack of motivation Unavailability of an open phone line or email Lack of commitment Lack of feedback and follow-up from NAFDAC Patient factors Lack of time and unavailability of the form Fear of indictment Reporting process Lack of documentation culture
Ways of improving ADR reporting among community pharmacist	Education, training, and awareness programs for both community pharmacists and the public Financial and non-financial motivations Increasing the availability and accessibility of the yellow form Feedback and follow up Learning from other countries Commitment Compulsion Simplifying the reporting process

### Barriers to ADR reporting among community pharmacists

Several barriers to ADR reporting were identified by the KIs. The most frequently cited barrier by almost all the participants was a lack of knowledge and awareness. Many KIs suggested that inadequate training of the CPs on the importance of ADR reporting and the reporting process hinders ADR reporting among CPs in the state. They also blamed poor reporting on this factor. One KI stated:

“The major issue is that community pharmacists are not well educated on ADR and the importance of reporting it. I feel that when they are trained on ADR and its importance, they will take it seriously. They have not created full awareness on ADR….” (PPF1).

“I think it is not lack of time. It is just awareness, education so that people will get to know the importance of reporting to NAFDAC for proper analysis….” (DPS2).

“I believe also that why people are not reporting is because they do not know the reporting process…….” (PA2).

“…. It is just awareness, education, and a lot of awareness….” (PCN1).

“……If they give us the form and tell us who to report to, and who to submit it to after reporting. I think it would make some people start….” (PAE3).

Secondly, most KIs suggested that a lack of motivation poses a problem to ADR reporting. They further explained that since most CPs are self-employed and do not depend on anybody for their salary, they are always focused on making money and might not easily allocate time to report ADRs unless there is motivation. An informant presented that:

“…. motivation by giving out something to motivate the pharmacists. NAFDAC rarely motivates people except for that period of Gentamycin 280 that they shared forms in community pharmacies, the only thing I see them doing is to work into a community pharmacy and look for drugs not registered in Nigeria….” (PPF3).

Another participant emphasized:

“…All these things, you have to sacrifice your time, your energy, and probably some of your money because you have to do some reporting. Community pharmacists pay themselves. So they are very much after how to earn money rather than how to do all that documentation on ADR or some other matters. Some financial aid will encourage participation…” (DPS2).

Similarly, a lack of commitment was mentioned as one of the reasons why ADR reporting is a challenge among CPs. One of the respondents further explained that since reporting ADRs is not mandatory, it requires commitment for one to report it.

“It is a big problem and it requires a sense of commitment. Something you do without thinking of getting any reward whatsoever but you know you are doing it for the betterment of the people and to enlighten the community too. Because there is no penalty for non-reporting. It requires a sense of commitment. That, I may say, is lacking because there is no motivation…” (PA1).

Yet, a few respondents suggested that the ADR reporting process including where, and how to access the reporting forms in addition to overwhelming reporting requirements hinders ADR reporting among CPs in the state.

“….. Simplifying the reporting, many times, the things you have to report are so big, they might never see all those things happening, but if we begin to do the least amount of reporting, we may come out better……” (PPT1).

The unavailability of an open phone line or email from NAFDAC was also suggested by some KIs as another factor that hinders ADR reporting.

“…..Sometimes even the number they put in that their yellow form is dead. There supposed to be an open phone line where you can report something and action should be taken….” (PPF4).

“…..On my own part too, at times I find it difficult to get this form, fill it and take it to NAFDAC, but assuming that they have a phone line or email that you can easily forward it to, it will make reporting better……” (PAE2).

An appreciable number of the KIs who claimed to have reported ADRs before complained about a lack of feedback and follow-up from NAFDAC as a factor that discouraged them from reporting more ADRs.

According to one of the respondents:

“…. I did the report once or twice but the issue is that there was no feedback, if you report and there is no feedback, how would you be sure you made an impact, so, it discourages one, but if there is feedback and follow-up and steps taken and they communicate you regularly. it will encourage you to do more. …” (PPT1).

“……I have, just once. I was disappointed because even the pharmacovigilance center that I reported to, I didn’t get any feedback from them. So that one too is another demoralizing factor…….” (PA1).

“……The only challenge is when you report and you don’t get feedback from the people you reported to, it mellows you down. I don’t think anybody has ever received feedback on ADRs……” (PAE1).

Patient factors including patients behaviours and reluctance to return to the original community pharmacy for follow-up were also mentioned as reasons why ADR reporting is a problem among CPs in the state. The respondent further explained that:

“……. Most patients, when they experience ADRs, do not come back to the CP that gave them the medication but rather, visit another pharmacy or even the hospital, who might mistake the ADR for another ailment entirely. Thus, such ADR might never be reported……” (PPT3).

Many KIs complained that a lack of time in addition to the unavailability of the form poses a barrier to ADR reporting. For instance, one KI described that;

“…. For Community pharmacists, maybe it is time and the way to go about it, I don’t think it is the duty of community pharmacists to start leaving their jobs to go to NAFDAC or to report to the manufacturer. NAFDAC especially those people in pharmacovigilance should share those forms monthly or quarterly and they should be able to go around and collect the form they can even liaise with the association and tell those who have seen any adverse reaction and report to come to the meeting with it and they will collect it from that point. So that they are saving everybody’s time…….” (PPT4).

Fear of indictment was also stated as one of the barriers to ADR reporting.

“….. People being afraid to report is another issue, if you report you might be indicting yourself because the drug distribution is not properly structured, Pharmacists get products from the wrong channel to survive and when you report NAFDAC says can I see where you bought it from or can I see the invoice and you cannot show it. You don’t want to embarrass yourself so you just keep quiet….” (PAE3).

In addition, the lack of documentation by CPs is another barrier to ADR reporting remarked by the PCN state officer. According to her, despite organizing a lot of training on documentation, the CPs are yet to inculcate the habit of documentation.

“We do a lot of training on documentation because the slogan is whenever it is not documented it was not done. We need our people (the pharmacists) to imbibe the habit of documentation because we find out that we don’t have data on so many things….” (PCN1).

Finally, the lack of a systematic approach to reporting, the increased workload of CPs, and the non-mandatory nature of ADR reporting is a problem among CPs.

### Ways of improving ADR reporting among community pharmacists

In suggesting ways of improving ADR reporting by community pharmacists in the state, nearly all respondents noted the need to conduct training on ADR reporting. Typical comments by the KIs were:

“…. I think there should be training for community pharmacists to help them know the possible things that are required in the reporting and the importance, where and how they will report it ….” (PRE1).

“…. Training is the most important thing because even some community pharmacist doesn’t even know (how to report). There should be a workshop by NAFDAC especially for the younger ones ……” (PAE4).

“….. the general public they are not fully aware of the procedure to do the reporting. Even though sometimes I have seen and heard the advert on reporting ADR on radio it is only a handful of people that can pick that information and act on it. There is still a need to keep creating an awareness in reporting adverse drug reactions………” (PA1).

A handful of KIs also recommended that awareness of ADR reporting should also be created in order to sensitize the CPs on the need to report ADRs. They also suggested various means by which it could be achieved. Their statements were:

“….. The National Pharmacovigilance unit of NAFDAC should create more awareness through radio jingles, training in conferences and workshops for pharmacists or any other healthcare professional involved or had anything to do with ADR reporting….” (PRE3).

“…. When we have community pharmacy meetings we should have a poster representation, an advert of the form given and it is showcased at the entry of the meeting point; reporting of adverse drug reaction by the pharmacist is a priority…”. (PAE1).

“…NAFDAC should also distribute their banner to each community pharmacy, pasting it in the office of the community pharmacist and it is also a representative to the patient; please report any ADR you have to this pharmacy. This is a level of awareness and advocacy…” (PAE1).

Several pharmacists suggested that the motivation of CPs would go a long way in improving ADR reporting. They stressed the need to motivate CPs either through monetary incentives or non-monetary means. A tax credit, waiver of PCN dues, and provision of certificates to appreciate reporting pharmacists were specifically mentioned. Their comments included:

“……If pharmacists are given some monetary incentives on improving adverse drug reaction maybe it will improve their reporting rate or create a form of motivation for reporting ADR……” (PPF3).

“…. Since the government has not done anything about it, The ACPN can champion it maybe grade it; and possibly if you report about 5 you will be recognized, 20, there will be a waiver on your annual due. All these can encourage people, when you know that you are doing something and you are getting a reward at the end there is that motivation…” (PA1).

“….. There supposed to be an incentive. Incentive does not mean monetary incentive; it can be a certificate to appreciate one on what he has done over the years….” (PAE1).

“……. There is a need for an incentive for example; you know that NAFDAC said that you must pay before they dispose of your expired drugs. if NAFDAC could say that if you report a certain number of reports they can dispose of your expired drugs without payment. This could improve ADR reporting in the state ……” (PPT1).

Again, making the forms accessible and distributing them to community pharmacists will enhance ADR reporting among CPs in the state. The respondents advocated for NAFDAC to take a step further by distributing the forms. One respondent noted:

“…. I think one of the ways is for NAFDAC to make sure that the form is dropped at every pharmacy premises so that people will get it because sometimes if you ask people, they will say where is the form. So if this form is available, people will easily report. NAFDAC also should go round to collect it ….” (DPS1).

Another respondent suggested that learning from other countries would help enhance ADR reporting in the state.

“…Nigeria is not in isolation from other countries. We know what is happening in other countries even Ghana, and Cameroon. If you have visited these places and you see what is happening in these countries, you will be forced to close down your practice. Things are organized over there; you have for instance within Anambra state a center that is responsible for ADR where you will forward your data because community pharmacists are expected to be producing data….” (PPT5).

Feedback and follow-up from NAFDAC were also noted as factors that would encourage ADR reporting.

“….. but if there is follow up and steps taken and they communicate with you regularly, it will encourage you to do more. …” (PPT1).

Commitment on the side of the CPs was also mentioned as a key to improving ADR reporting in the state.

“..… There is no other way than their own devotion, commitment, and interest. They have to read to know what ADRs are all about, how to detect them, how to do the questioning, how to lure your patients so that they can really give you all the answers they need….” (DPS2).

One of the KIs suggested that ADR reporting be made mandatory and linked to annual registration. She concluded that this is the only basis that CPs will be able to report. She noted:

“…. Let it be linked to their registration, without any compulsion they would not do it….” (PSMoH3).

“…. Also things are now automated, why do we have to fill this form manually? It should be an online thing like an E-form, where you just fill it and automatically it goes. Meanwhile, the form should be mobile phone-friendly. Telling us to fill a document that nobody comes to collect or you will now fill and send it to the NAFDAC office yourself. I don’t buy that idea….” (PA3).

“….. It is also possible to have a phone communication; a phone line that when people have ADR they can call immediately, calling and responding quickly….” (PAE1).

“…. It is good for us to have this particular NAFDAC WhatsApp number that we can just snap the picture of the form you have filled and send or an email specifically for that ADR issue…” (PAE2).

## Discussion

This study sheds light on the views of community pharmacists’ stakeholders regarding ADR reporting trends and strategies to enhance it in Anambra state, Nigeria. While the participants demonstrated a theoretical understanding of ADR reporting importance, the practical application seemed lacking. This points to a potential gap between theoretical knowledge and the practical skills required for effective reporting.

The study emphasized the pivotal role of community pharmacists in ADR reporting, given their accessibility to patients^31^. The CPs are well placed to promote and support patient safety and discuss any adverse reactions patients are experiencing^32,33^. This accessibility positions them as crucial contributors to meeting the WHO's target of approximately 200 ADR reports per million inhabitants per year which is a benchmark for adequate reporting across various regions^18^.

Although the knowledge level of the interviewed community pharmacists regarding pharmacovigilance was high, it appeared more theoretical than practical. This could be seen from their poorly expressed reporting involvement despite their high pharmacovigilance knowledge. This suggests that while they may have acquired the theoretical knowledge from formal education, practical skills may not have been developed. ADR reporting is a skill that involves some amount of physical coordination, or experiential learning that is mainly acquired through practice^32^. Hands-on training and experiential learning were recommended to bridge this gap, aligning with studies advocating for practical demonstrations in ADR reporting training^34^.

ADR reporting is a call to duty and commitment to patient safety^35^, with the reporting personnel being a source of data on medicinal products' safety and new risk identification^33^. Although a few of the key informants had reported ADR in the past, underreporting emerged as a significant issue among the participants. The reasons provided, including negligence, distance barriers, and litigation concerns, highlighted potential misconceptions about ADR reporting. For instance, in addition to obtaining and returning ADR forms from NAFDAC state offices, as well as NAFDAC headquarters in Abuja, and the zonal Pharmacovigilance offices across the six geopolitical zones of the country, some of the informants were unaware of the availability of an online ADR e-reporting form^36^, which, ideally, should eliminate the distance barrier. This highlights the need for advocacy and regular updates for practitioners on reporting procedures and their importance.

Feedback and follow-up from NAFDAC were identified as crucial factors that could motivate pharmacists to report ADRs. This aligns with a previous study highlighting the need for effective communication channels between reporting pharmacists and regulatory agencies^19^.

The study also revealed various other barriers to ADR reporting including lack of time, unavailability of reporting forms, fear of indictment, and increased workload. These barriers are also consistent with findings from similar studies^15,21,28,37–39^ emphasizing their widespread relevance.

Training and awareness were identified as key strategies to improve ADR reporting in the state. This is consistent with a similar study in other regions that advocated for further education and training on ADR reporting^37,40^. Additionally, creating awareness through mediums like radio jingles, workshops, conferences, and posters could also be used. These strategies aim to educate both pharmacists and the public on the importance of ADR reporting.

Incentivization emerged as a potential motivator for pharmacists to engage in ADR reporting. Few studies have assessed the role of small financial incentives for reporting ADR and revealed that this led to an increase in reported numbers^41^. However, the use of this approach must be applied with caution to avoid incentive-induced reporting that may result in either the reporting of trivial cases or non-reporting at all as had previously presented^42^. While financial incentives were mentioned, non-monetary rewards such as reduced regulatory dues and recognition through awards were also suggested. This multifaceted approach to motivation may cater to a broader range of pharmacists' preferences.

The study highlighted the need for feedback and follow-up mechanisms, simplification of reporting processes, and increased commitment from pharmacists. Additionally, making ADR reporting mandatory and linking it to license renewal was proposed as a way to ensure compliance.

This aligns with a UK study^37^ where half of the respondents believed that making ADR reporting mandatory is essential for enhancing patient safety.

## Limitations

This study acknowledges several limitations that may have influenced the findings in this work. Firstly, the use of structured interviews can be susceptible to recall bias as participants may not accurately remember some specific details. However, the researchers attempted to mitigate this by triangulating information from diverse informants. Secondly, as key informants with extensive knowledge of the subject, there is a potential for them to disagree with the main topic. The researchers who were well-versed in the field, made efforts to keep the interviews focused. Another limitation is that the study focused solely on key informants in Anambra state, and did not gather information from other states in Nigeria. Including informants from other regions could provide a broader perspective on improving ADR reporting. Hence, we propose that a study involving participants from various states would be beneficial in providing a more comprehensive understanding of the strategies for enhancing ADR reporting. Additionally, the construction of the interview guide was not guided by a behavioural framework, which may have limited the research findings.

## Conclusion

The study highlights a low level of community pharmacists' involvement in reporting ADRs in Anambra state. It suggests that training and awareness programs, among other strategies, could significantly enhance ADR reporting among community pharmacists. The study calls for further research to explore and implement these approaches. Additionally, the study advocates for regular public education campaigns, including radio jingles and workshops, to increase awareness of ADR reporting. It also recommends strengthening the pharmacovigilance unit of NAFDAC to enable effective follow-up on reported ADRs, ultimately encouraging ADR reporting in the country.

### Supplementary Information

Supplementary Information.

## Data Availability

The data underlying this article cannot be shared publicly due to the privacy of individuals who participated in the study. The data will be shared on reasonable request with the corresponding author. Access to this study data is restricted due to ethical issues.
