# Supplementary material for: Identifying strategies to improve adverse drug reporting through key informant interviews among community pharmacists in a developing country
Source: Sci Rep. 2024 Jul 22;14:16821. doi: 10.1038/s41598-024-67263-8 (PMC11263357; doi:10.1038/s41598-024-67263-8)
Supplement: Supplementary file 1 — Supplementary Information. [file 41598_2024_67263_MOESM1_ESM.docx]

**File 1: Questionnaire**

**Semi-structured Interview guide**

| **Questions** | **Answers** |
| --- | --- |
| **Adverse Drug reaction reporting** |  |
| What is your understanding of ADR and whose role should it be?   - Probe physicians? - Probe Pharmacists or nurses? |  |
| Who in particular do you think should be more involved in reporting ADR? |  |
| In the case of pharmacists, which among the pharmacists should be more involved? |  |
| How would you rate community pharmacists’ involvement in reporting ADR in Anambra state?  Probe   - On a scale of 0-5 rate their level of involvement. - Where,0- No involvement   5- Maximal involvement |  |
| Have you ever reported ADRs?   - If Yes, how many times have you reported? - If No, why? - Is it that you don’t know how to report or you have not been trained? |  |
| How is ADR reported in Nigeria?   - Have you seen an ADR reporting form and what does it look like? - How is the form accessed? - Who is in charge of its collection in Nigeria - Where and how do you submit it? |  |
| **Barriers to ADR reporting among community Pharmacists** |  |
| In your opinion, why do you think it is difficult for community pharmacists to report ADRs or why are community pharmacists not participating in ADR reporting in the state?  Probe   - Ignorance of what, how, and whom to report to? - Lack of time? - Lack of incentives? - Lack of interest? - Unavailability of the yellow cards for reporting? |  |
| What are the challenges that you have encountered in reporting ADRs? |  |
| **Possible ways and means of improving ADR reporting by community pharmacists** |  |
| What interventions have been made so far by the authorities/ your office/ others in increasing ADR reporting among CPs in the state?  Probe   - Has any training been conducted so far on ADR reporting in the state? - Have incentives been offered as a means of increasing ADR reporting in the states? - Has there been any scenario where yellow cards were made accessible to community pharmacists? |  |
| In your opinion, what are the possible ways and means by which ADR reporting could be improved among community pharmacists in the state? |  |
| Do you have any questions? |  |

Thank you for the information and your time.
